# Hippo Signaling Pathway in Pancreas Development

**DOI:** 10.3389/fcell.2021.663906

**Published:** 2021-05-17

**Authors:** Yifan Wu, Pauline Aegerter, Michael Nipper, Logan Ramjit, Jun Liu, Pei Wang

**Affiliations:** ^1^Department of Cell Systems and Anatomy, The University of Texas Health San Antonio, San Antonio, TX, United States; ^2^Department of Obstetrics, The Second Xiangya Hospital, Central South University, Changsha, China

**Keywords:** Hippo, pancreas, development, Mst1/2, LATS1/2, YAP

## Abstract

The Hippo signaling pathway is a vital regulator of pancreatic development and homeostasis, directing cell fate decisions, morphogenesis, and adult pancreatic cellular plasticity. Through loss-of-function research, Hippo signaling has been found to play key roles in maintaining the proper balance between progenitor cell renewal, proliferation, and differentiation in pancreatic organogenesis. Other studies suggest that overactivation of YAP, a downstream effector of the pathway, promotes ductal cell development and suppresses endocrine cell fate specification via repression of Ngn3. After birth, disruptions in Hippo signaling have been found to lead to de-differentiation of acinar cells and pancreatitis-like phenotype. Further, Hippo signaling directs pancreatic morphogenesis by ensuring proper cell polarization and branching. Despite these findings, the mechanisms through which Hippo governs cell differentiation and pancreatic architecture are yet to be fully understood. Here, we review recent studies of Hippo functions in pancreatic development, including its crosstalk with NOTCH, WNT/β-catenin, and PI3K/Akt/mTOR signaling pathways.

## Introduction

Hippo signaling plays an important role in regulating cell proliferation and apoptosis; thus, it is thought to be a central regulator of organ size and tissue homeostasis. A complex network of biological processes has been shown to regulate the Hippo signaling pathway, including determinants of cell polarity and cell-cell junctions, factors mediating the activation of Hippo kinases, mechanotransduction, soluble factors acting through G-protein-coupled receptors (GPCRs) and Rho GTPases, and metabolic pathways mediating YAP/TAZ nuclear localization (mevalonate pathway) or their binding to TEAD factors (glucose metabolism and aerobic glycolysis) (Maugeri-Sacca and De Maria, 2018). These pathways are all important for organogenesis, asserting the essential roles of the Hippo pathway in the development of an organism (Zheng and Pan, 2019). Defective Hippo signaling can lead to pancreatic dysfunction such as defects in both embryogenesis and postnatal development. This review aims to outline the roles of the Hippo signaling pathway in pancreatic development.

The pancreatic developmental process begins with two outpouchings arising from the distal foregut endoderm, forming into the dorsal and ventral pancreatic buds. Meanwhile, the organ must properly form acinar cells, ductal cells, and five different endocrine cell lineages. Pancreatic tubulogenesis is a complex process that must be regulated by a network of transcription factors and signaling pathways (Jennings et al., 2015; Fujitani, 2017). As an essential organ for metabolic regulation, dysfunction of the endocrine pancreas causes serious chronic metabolic diseases, such as diabetes mellitus. On the other hand, damage to the exocrine pancreas can lead to pancreatitis and pancreatic cancer. Possible treatment strategies for these debilitating diseases include the generation and transplantation of pancreatic endocrine cells, reprogramming the original pancreatic cells (Zhou and Melton, 2018), or reducing inflammation to subsequently revert metaplastic cells back to normal cells. Understanding how the pancreas develops with correct organ size, composition, and architecture will provide insights into these diseases. In this review, we summarize recent knowledge of the role of Hippo signaling in pancreatic development, discuss the importance of crosstalk with other signaling pathways, and explore future directions of investigation.

## An Outline of Pancreatic Development

The mammalian pancreas is a dual-function organ that is essential for proper digestion and subsequent energy consumption. The exocrine pancreas is known to produce digestive enzymes (lipases, proteinases, and amylases), which are secreted by acinar cells and delivered to the small intestine by a branched ductal network. The endocrine pancreas is key to the maintenance of glucose homeostasis through five kinds of hormone-producing cells (Shih et al., 2013). These endocrine cells cluster in the islets of Langerhans and include α-, β-, δ-, PP-, and ϵ- cells that synthesize glucagon, insulin, somatostatin, pancreatic polypeptide, and ghrelin, respectively. Pancreatic development has been reviewed in detail (Benitez et al., 2012; Bastidas-Ponce et al., 2017; Larsen and Grapin-Botton, 2017). Here, we provide a brief review of pancreatic development to help contextualize the role of Hippo pathway in it.

**FIGURE 1:**
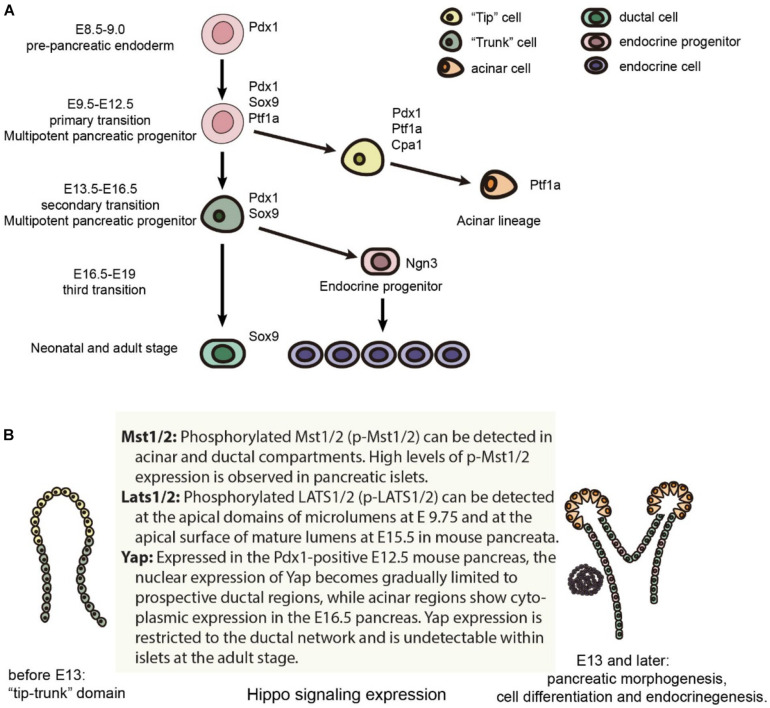
Mouse pancreas development and the expression of Hippo components. **(A)** Major cell lineages in the pancreas and the master regulators of each lineage. **(B)** The expression of Hippo components in the mouse pancreas.

Early pancreatic organogenesis can be divided into two transitional stages: the primary transition occurs during E9.0-E12.5 while the secondary transition occurs during E13.5-E16.5 (Figure 1A; Bastidas-Ponce et al., 2017). The primary transition is marked by the specification and proliferation of pancreatic progenitors, accompanied by some development of glucagon-producing cells. Importantly, the number of multipotent pancreatic progenitors developed during the primary transition correlates with final organ size (Seymour, 2014). Prior to the onset of the primary transition (E8.5-E9.0), *Pdx1* (pancreatic and duodenal homeobox 1) expression marks the pre-pancreatic endoderm. In a *Pdx1*-null pancreas, the dorsal bud develops normally until approximately E10.5, is smaller by E11.0, and fails to grow past E11.5, while the ventral bud never invaginates (Hale et al., 2005). *Pdx1* also regulates pancreatic tubulogenesis and E-cadherin expression (Marty-Santos and Cleaver, 2016). Lastly, *Pdx1* can negatively regulate the expression of pancreatic ductal cell-specific keratin 19, which leads to the inhibition of the ductal differentiation program within the pancreatic endocrine compartment, particularly in β cells (Deramaudt et al., 2006). Because of these influential roles, *Pdx1* is often termed a “master regulator” of whole pancreatic development (Figure 1A; Vinogradova and Sverdlov, 2017).

Specification and differentiation of pancreatic cells during the primary transition is further guided by a complex network of additional transcription factors (Arda et al., 2013). One such transcription factor, *Ptf1a*, is expressed in the early pancreas, directing the expansion of multipotent progenitor cells (MPCs) along with the specification of acinar cells, and is finally restricted to acinar cells (Jin and Xiang, 2019). *Sox9*, which is also required for maintenance of the pancreatic progenitor cell pool, is critical in gene regulation as well (Seymour et al., 2007; Seymour, 2014). Within the human fetal pancreas, *Sox9* is important for the expression of *Ngn3* and other molecular markers of endocrine cell differentiation (McDonald et al., 2012).

During the secondary transition of pancreatic organogenesis, all five hormone-producing endocrine cells begin to develop, and amylase-producing acinar cells arise from the extending tip epithelium. During this transition, basic helix-loop-helix transcription factor neurogenin 3 (*Ngn3*) drives bipotent pancreatic progenitor cells toward the endocrine cell fate (Gradwohl et al., 2000; McGrath et al., 2015). Furthermore, biphasic expression of *Ngn3* correlates with the “first” and “second” transitions, which encompass two distinct waves of embryonic endocrine differentiation (Villasenor et al., 2008). Not only is *Ngn3* a master regulator of pancreatic islet differentiation and regeneration, but it also initiates stepwise delamination of differentiating endocrine cells during pancreatic development (Rukstalis and Habener, 2009; Gouzi et al., 2011). Lastly, achieving high levels of *Ngn3* expression is a critical step for endocrine commitment from multipotent pancreatic progenitors (Wang et al., 2010; Magenheim et al., 2011).

A “third” transition has been proposed by some researchers, which occurs from E16.5-E19. During this time, endocrine cells migrate and cluster into islets of Langerhans while acinar cells further expand. However, the signaling pathways guiding the formation of the islets of Langerhans are not fully understood. Starting from the secondary transition, committed endocrine cells leave the ductal epithelium, migrate into the surrounding mesenchyme to coalesce into proto-islets, and finally develop into functional islets of Langerhans. The process is regulated by the spatiotemporal activities of various signaling factors along with coordinated cell dynamics, such as the crosstalk between endothelial and mesenchymal cells (Bastidas-Ponce et al., 2017).

As β cells exit the epithelial progenitor cell layer, they acquire some mesenchymal characteristics (Cole et al., 2009). Cdc42-mediated tubulogenesis controls cell specification by providing the correct micro-environment, and it links actin dynamics to pancreatic β cell delamination and differentiation (Kesavan et al., 2009; Kesavan et al., 2014). Snail2/Slug, a known inducer of epithelial to mesenchymal transition (EMT) and cell movement, plays a vital role in endocrine cell delamination and migration as well (Rukstalis and Habener, 2007; Lee et al., 2011). The transcription co-repressor Grg3/Tle3 promotes the delamination of endocrine progenitors along with β cell differentiation (Morris and Machesky, 2015). During β cell development, EphB3 can mark delaminating endocrine progenitors and help define the timeframe of endocrine differentiation (Villasenor et al., 2012).

Besides the formation of different cell types, the developmental mechanisms controlling the morphogenesis of the pancreatic epithelium are also essential (Shih et al., 2013). When the pancreatic buds develop, their morphogenesis generates a highly branched, tree-like tubular epithelial network. The process includes epithelial stratification, cell polarization, microlumen formation and fusion, and finally gives rise to a luminal plexus to be remodeled into a complex network. From E11.5 onward, the mouse pancreatic epithelium consists of MPCs that progressively segregate into tip or trunk domains and are allocated to acinar or bipotent endocrine/duct progenitor cell fates, respectively (Larsen and Grapin-Botton, 2017). The cells in the tip domain express Ptf1a and Nr5a2, while the trunk cells express Sox9, Nkx6.1, Hnf1b, and Pdx1. Several signaling pathways such as Notch, EGFR and RhoA regulate the tip-trunk pattern along with tubulogenesis (Murtaugh et al., 2003; Afelik et al., 2012; Azizoglu et al., 2017; Lof-Ohlin et al., 2017). Over the past decade, efforts have aimed to pinpoint the molecular mechanisms governing pancreas development and organogenesis. However, much remains to be discovered, particularly regarding the cellular processes that coordinate the morphogenesis of this complex organ (Bastidas-Ponce et al., 2017).

## An Overview of the Hippo Signaling Cascade

The Hippo signaling pathway is a highly conserved kinase cascade that was initially characterized in *Drosophila*, and most components have since been found to have multiple orthologs in mammals. The pathway regulates diverse cellular processes, including proliferation, differentiation, cell survival, and organ size (Gomez et al., 2014; Mo et al., 2014). In mammals, the Hippo pathway consists of a kinase cascade of the Ste-20-like protein kinases MST1 and 2, which phosphorylate large tumor suppressor 1 and 2 (LATS1/2). Once phosphorylated, LATS1/2 are activated and can phosphorylate the main effectors of the Hippo pathway, the transcription coactivators Yes-Associated Protein 1 (YAP), and Transcription co-activator with a PDZ-binding motif (TAZ). Following phosphorylation, YAP and TAZ are either sequestered in the cytoplasm or degraded in a ubiquitin-proteasome-dependent manner. When LATS are inactive, unphosphorylated YAP and TAZ translocate to the nucleus to initiate transcription and induce the expression of genes regulating proliferation, differentiation, and apoptosis. Although YAP and TAZ have some redundant functions, studies of mammary and kidney organogenesis have shown that the inactivation of either gene produces very different phenotypes (Piccolo et al., 2014; Skibinski et al., 2014), emphasizing their distinct roles and the need to further delineate their functions in other tissues.

Neither YAP nor TAZ have a DNA-binding domain, so they require other transcription factors to exert control on gene expression. Transcription enhancer associate domain (TEAD) 1-4 have been shown to interact with YAP and TAZ to mediate downstream gene expression (Santucci et al., 2015), including genes involved in cell growth and proliferation (e.g., DNA replication, mitosis, and chromosome organization) as well as stem cell identity and tissue architecture (e.g., cytoskeleton, extracellular matrix) genes (Holden and Cunningham, 2018). The various target genes of YAP-TEAD result in a diverse set of functions of the Hippo signaling pathway including development, organ size control, tissue homeostasis, cell ploidy, innate immunity, miRNA biogenesis, atherogenesis, and tumorigenesis (Kim and Jho, 2018).

A number of studies have revealed that the Hippo pathway is a critical player in the development of many different organ systems, including the heart, lung, brain, and liver (Dai et al., 2017; Wu et al., 2017; Nantie et al., 2018; Wang et al., 2018; Noce et al., 2019; van Soldt et al., 2019). These studies indicate that the Hippo pathway has distinct functions under different physiological and pathological conditions, highlighting the importance of investigating this pathway in a tissue- and context-specific manner. Thus, the Hippo signaling pathway and its transcription effectors YAP/TAZ have emerged as key regulators of numerous developmental decision-making processes.

## Recent Advances in Understanding the Hippo Pathway in Pancreas Development

Recent studies have identified that the Hippo signaling pathway and its effectors are vital for pancreatic development and function. It influences not only embryonic pancreatic development but also several pancreatic diseases, including acute and chronic pancreatitis, pancreatic cancer, and diabetes mellitus. Multiple Hippo pathway genes have been shown to be expressed in the developing pancreas (Figure 1B). These genes have since been investigated through selective deletion at embryonic and adult stages using different pancreatic-specific Cre lines in genetically engineered mice models (George et al., 2012; Gao et al., 2013; Braitsch et al., 2019; Liu et al., 2019). Some Hippo components have also been investigated in human pancreatic cells differentiated from human pluripotent stem cells (Zhang et al., 2013; Cebola et al., 2015; George et al., 2015; Mamidi et al., 2018; Rosado-Olivieri et al., 2019).

Utilizing genetically engineered mouse models (GEMM) is the primary method by which various components of the Hippo pathway have been studied within the context of pancreatic development. It is also essential to use specific Cre or Cre-ER lines to achieve pancreas-specific gene modification with spatial and temporal control. There are over seventy pancreatic Cre driver lines directed by over thirty different gene promoters (Magnuson and Osipovich, 2013). For example, 11 Cre or Cre-ER driver lines have been generated using different DNA fragments of the Pdx1 promoter, including Pdx1^*Cre*–*early*^ and Pdx1^*Cre*–*late*^, which enable Cre to be expressed at different time points during mouse pancreatic development. Thus, we must consider the specific Cre driver lines utilized when discussing knockout studies of Hippo components and their resulting phenotypes.

### MST: The Mammalian Ste20-Like Kinases 1 and 2

MST1 and 2 kinases were the first components of the Hippo pathway to be investigated in pancreatic development. Phosphorylated Mst1/2 (p-Mst1/2) can be detected in the acinar and ductal compartments of a developing mouse pancreas, and much higher levels of p-Mst1/2 expression are found in the pancreatic islets (George et al., 2012). A *Cre/loxp* system was used to delete Mst 1 and 2 (Mst1/2) within the pancreatic epithelium, (*Pdx1^*cre*^Mst1^–/–^Mst2^*fl/fl*^*, DKO) and (*Pdx1^*cre*^Mst1^*fl/fl*^ Mst2^*fl/fl*^*, DKO) (George et al., 2012; Gao et al., 2013) in early development. The Pdx1 Cre lines used in these two studies were notably different; Gao et al. (2013) utilized *Pdx1^*early*^* while the *Pdx1^*Cre*^* line that (George et al., 2012) used has not been characterized in detail. Nonetheless, the major resulting phenotypes were similar: de-differentiation of acinar cells to ductal-like structures, immune infiltration, and auto-digestion. Typical characteristics of pancreatitis were also observed postnatally. Although pancreatitis phenotypes occurred early in *Mst1/2* null mice (around P14), tumor formation was not observed at 1 year of age (George et al., 2012).

Although the phenotypes found from these two papers were similar, different mechanisms for the results are discussed. The first difference surrounds the paradox as to how increased cell proliferation within the exocrine cells is associated with reduced pancreatic mass. Gao et al. (2013) suggest that the loss of *Mst1/2* and subsequent de-differentiation of acinar cells triggers an upregulation in cell death. Conversely, George et al. (2012) found that there are no changes in cell apoptosis, and they propose that the reduction in pancreatic mass is instead the result of activation of auto-digestion. Specifically, *Mst1/2* DKO mice fail to form a highly branched ductal network, which causes digestive enzymes to be released into the surrounding tissue and initiate auto-digestion. In addition, Gao et al. (2013) propose that acinar cell development is not affected by the loss of *Mst1/2*, because acinar-related transcription factors, Mist1, Ptf1a, RBP-JL, and Lrh1, are found to have normal expression at P0. The two papers also consider different reasons for immune infiltration. Gao et al. (2013) suggest that acinar de-differentiation precedes cell death and pancreatitis, and that loss of *Mst1/2* promotes leukocyte invasion. George et al. (2012) report that acinar to ductal metaplasia is the result of immune infiltration, but they do not propose the molecular mechanisms for the initial onset of pancreatitis.

The second difference between the two papers is in regard to the endocrine compartment. Gao et al. (2013) found that the loss of *Mst1/2* leads to an increase in the ratio of α/β cells. George et al. (2012) reported changes to the islet architecture in that β cells were not surrounded by α cells, but the α/β ratio stayed the same. Numerous single insulin positive cells were found throughout the Mst1/2 KO pancreas instead of the normal pattern of central β cells surrounded by α cells. Neither of these two papers reported precise time points during the discussion of endocrine cell development. Despite these differences, they both conclude that the overall function of the islets is not affected by the loss of *Mst1/2*. Both papers also demonstrate that the expression of YAP is undetectable in endocrine cells, even in the DKO offspring from E16.5. This suggests that YAP is regulated in a Hippo-independent manner during pancreatic endocrine development.

Through analysis of gene ontology of differentially expressed genes in DKO pancreas versus control, Gao et al. (2013) suggest that the Hippo pathway may first lead to changes in cell shape and adhesion, followed by effects on cellular identity. In their *Mst1/2* DKO mice model, the pancreas exhibited deregulation of genes involved in integrin signaling and cell adhesion (Gao et al., 2013). Although both studies display YAP stabilization through low levels of YAP phosphorylation in DKO mice, only Gao et al. (2013) used a genetic method to demonstrate that the phenotype of DKO pancreas was largely rescued by deleting one copy of the YAP gene. They also showed that overexpression of a constitutively activated form of YAP in the pancreas mimics the *Mst1/2* null phenotype. Altogether, these papers delineate the functions of MST1/2 in pancreatic development and suggest that YAP is the downstream factor responsible for the pancreatic defects (George et al., 2012; Gao et al., 2013).

### LATS: Large Tumor Suppressor 1 and 2

Pancreatic progenitor epithelial cells give rise to acinar, ductal, and endocrine lineages, coinciding with branching and tubule development. The process of organ morphogenesis includes epithelial stratification, cell polarization, microlumen formation and fusion, and formation of a luminal plexus to be remodeled into a complex tubular network. It is a complicated developmental process that requires multiple transcription factors and signaling pathways to govern it. However, we still do not have a complete understanding of how these processes are carried out. Nonetheless, it has been proposed that LATS1/2, the downstream kinases of MST1/2, function in pancreatic morphogenesis.

In the early developmental stage, phospho-LATS1/2 localize to the apical domains adjacent to mucin-1^+^ (MUC1^+^) microlumens in the normal pancreatic bud at E9.75, and are expressed at the apical surface of mature lumens at E15.5 (Braitsch et al., 2019). Following the early embryonic deletion of *Lats1/2* using Pdx1^*early*^ Cre (Gu et al., 2002), the pancreas lacks all differentiated cell types including the acinar, ductal and endocrine cells. The loss of pancreatic cell identity occurs before and during the secondary transition. At E10.75, there are no changes in expression levels of PDX1 and PROX1, but there are fewer NGN3^+^ cells. Knockout pancreata do not initiate branch formation at E11.5, and MUC1 expression is reduced at E12.5, which leads to the loss of cell polarity and subsequent failure of epithelial expansion/branching. Progressive luminal hyperfusion, increased lumen size, and loss of the normal ductal plexus have also been observed. When studying cell shape, increases in the ratio of apical to basal width leads to failure of apical constriction, which is vital for microlumen formation. Abnormal expression of Vimentin within epithelial cells can also be found at E11.5. However, the cells remain E-cadherin positive, suggesting incomplete EMT. Partial EMT is indicated by unchanged expression of most EMT transcription factors. At E13.5, the mutant pancreas remains small and rounded, which shows that early pancreatic morphogenesis requires a properly functioning Hippo signaling pathway.

**FIGURE 2:**
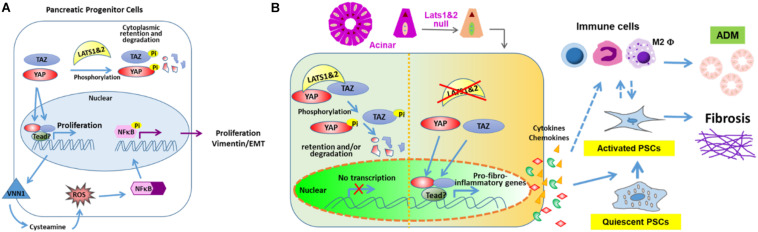
LATS 1 and 2 function in pancreas. **(A)** LATS 1/2 phosphorylate YAP/TAZ to indirectly suppress NFκB and aberrant EMT initiation to allow proper pancreatic morphogenesis. **(B)** LATS 1/2 control the intrinsic fibroinflammatory program in adult pancreatic acinar cells through inhibition of YAP/TAZ.

It was found that further deleting *Yap1&Taz* in *Lats1/2* null mice (*Pdx1Cre^*early*^; Lats1^*f*/*f*^; Lats2^*f*/*f*^; Yap1^*f*/*f*^; Taz^*f*/*wt*^*) largely rescues the phenotype, suggesting that LATS1/2 regulate pancreatic differentiation via YAP1&TAZ (Braitsch et al., 2019). It was found that loss of *Lats1/2* upregulates the expression of *Vnn1*, which leads to activation of ROS and NFκB signaling pathways. However, blocking the NFκB signaling pathway does not rescue the defects of pancreatic cell differentiation, indicating that activation of the NFκB pathway may be a secondary effect. It is suggested that LATS1/2 suppress the activation of NFκB and EMT via YAP (Figure 2A). Lastly, RNA-seq analysis indicates that cell adhesion molecules and tight junction pathways are upregulated, which may be a compensatory response to the defects of EMT, loss of apicobasal polarity, and altered cell shape (Braitsch et al., 2019).

Interestingly, the deletion of *Lats1/2* in adult pancreata has shown very distinct phenotypes (Liu et al., 2019). Our lab used the *Ptf1a*^*Cre*–*ER*^ system to delete *Lats1/2* specifically in acinar cells at the adult stage. The knockout mice displayed a very severe pancreatitis-like phenotype, which differs from the phenotype seen in both *Mst1/2* knockout studies. Subsequent analysis found that deletion of *Lats1/2* did not directly affect acinar proliferation and apoptosis. Instead, *Lats1/2* null acinar cells produced cytokines, such as CTGF and SPP1, which directly activated pancreatic stellate cells and consequently induced fibrosis and immune cell infiltration. This research suggests that Hippo signaling plays critical roles in acinar-stromal communication, which promotes the proliferation and metaplasia of acinar cells. Additionally, the phenotype can be rescued by further deletion of YAP and TAZ. These findings underscore the mechanism through which epithelial acinar cells can mount an inflammatory response within the pancreas (Liu et al., 2019). Disruption of Hippo signaling directly contributes to the activation of stromal cells through upregulation of inflammation and fibrosis-associated genes in acinar cells (Figure 2B). However, it will be important to fully explore the direct targets of YAP/TAZ and their contributions to the immune cell recruitment.

Developmental-stage deletion of *Mst1/2* produces a phenotype similar to what is seen in an adult-stage deletion of *Lats1/2*, but differs from a developmental-stage deletion of *Lats1/2*, even though both used same Pdx1^*early*^ Cre (George et al., 2012; Gao et al., 2013; Braitsch et al., 2019). The different phenotypes in these two knockout mice models suggest that, during pancreatic development, LATS1/2 may have other upstream regulators such as GPCR signaling, AMPK, or MAP4K (Hong et al., 2016), and further study will be required to reveal their identities. Although MST1/2 have yet to be deleted in adult acinar cells, the results will most likely phenocopy what has been seen in a LATS1/2 deletion, as it should also result in YAP/TAZ hyper-activation. Nevertheless, both MST1/2 and LATS1/2 affect pancreas development through YAP, making YAP the center of Hippo signaling pathway during development.

### YAP/TAZ

In the developing pancreas, progenitors show high levels of proliferation, followed by exiting the cell cycle and differentiating into different cell types. Here, it is important to maintain a balance between self-renewal and specification of daughter cells. It has been thought that the Hippo pathway contributes to this balance and overall organogenesis by regulating growth and the time at which progenitors exit the cell cycle (Halder and Johnson, 2011). As one of the most important effectors of the Hippo signaling pathway, YAP expression is robust, which coincides with the high levels of proliferation during the primary and secondary transition stages (George et al., 2015). However, in the pancreatic endocrine lineage, YAP expression is turned off at the RNA level rather than by the canonical Hippo pathway control, and the downregulation of YAP expression consequently correlates with a decrease in endocrine cell proliferation (George et al., 2015). Zhang et al. report that YAP1 is a target gene of miR-375, which acts on the 3’ UTR of YAP1 mRNA to decrease its mRNA and protein levels. Similar to silencing YAP1 by shRNA, the proliferation of pancreatic progenitor cells is inhibited significantly by forced expression of miR-375 (Zhang et al., 2013). Whether the suppression of YAP/TAZ in endocrine lineage cells is through microRNA or epigenetic regulation requires further investigation.

During early pancreatic development, the two outpouchings of distal foregut endoderm consist of multipotent epithelial progenitors that branch and specify into the trunk domain of bipotent pancreatic progenitors (bi-PPs), which further differentiate into both duct and endocrine lineages, and to the tip domains that give rise to acinar cells. YAP transgenic mice models (*tet-YAP1^*S*127A^;Pdx1^*tTA/*+^*, referred as YAPtg) in which constitutively active human YAP1 is expressed at E12.5 (Mamidi et al., 2018) have shown that YAP1 target genes including Cdc20, Ctgf, Birc5, and Snai2, are upregulated during pancreatic development. The expression of SOX9, HNF1β, MUC1, and E-cadherin are also upregulated, suggesting that overexpression of YAP drives bi-PP cells toward ductal lineage *in vivo*. Meanwhile, the expression of pancreatic progenitor markers (*Pdx1* and *Nkx6.1*), acinar markers (*Cpa1* and *Amy2A5*) and endocrine markers (*Ngn3* and *Ins1*) are lower in the YAP1tg embryonic pancreas compared with controls. In addition, normal branching and tip-trunk patterning is perturbed at E12.5. Altogether, the phenotypes indicate that YAP1 regulation is important for mid-embryonic pancreas development (Mamidi et al., 2018).

There has been an increase in research focused on identifying the extrinsic and intrinsic signaling mechanisms that govern cell fate, with activation of the Hippo signaling pathway occurring via sensors of cell density (Mamidi et al., 2018). Specifically, the F-actin-YAP1-NOTCH mechanosignaling axis, which ultimately controls the fate of bipotent pancreatic progenitors, is initiated by the interaction of the extracellular matrix with integrin α5. Cell spreading positively regulates YAP1 activity by forming actin bundles, which then drives progenitors toward a ductal fate. Conversely, endocrine specification is driven by cell confinement negatively regulating YAP1, dissociating actin bundles (Mamidi et al., 2018). Altogether, the Hippo signaling pathway helps to maintain the state of progenitors and promotes development of ductal lineage during the second transition in a YAP-dependent manner.

At E16.5, YAP becomes gradually limited to prospective ductal and acinar regions. Conversely, endocrine cells lack detectible YAP expression (George et al., 2015). The loss of YAP in endocrine development is independent of canonical Hippo signaling, and the regulation occurs at the transcription level. The reduction of YAP mRNA and proteins coincides with decreased proliferation of endocrine cells (George et al., 2012).

There are two possible mechanisms to explain how the Hippo signaling pathway drives cell proliferation. The first mechanism proposes that Hippo signaling regulates cell proliferation and apoptosis via a YAP-TEAD complex, which may directly activate genes governing proliferation. Using human embryonic pancreata and embryonic-stem-cell-derived progenitors, the regulatory landscape of *in vivo* and *in vitro* MPCs has been investigated (Cebola et al., 2015). Through analysis of RNA-seq and ChIP-seq data, it has been found that TEAD factors are vital components of the combination of transcription factors that activates both stage and lineage-specific pancreatic MPC enhancers. Additionally, TEAD1 is a core component of pancreatic progenitor cis-regulatory modules (CRMs). The role of TEAD and YAP in pancreatic development was determined using chemical and genetic inhibitors to disrupt TEAD/YAP complexes, which subsequently led to the reduction of pancreatic epithelial proliferation. These findings indicate that YAP-TEAD directly regulate the outgrowth of pancreatic progenitors. During these experiments, SOX9 was the proliferative mediator of TEAD and YAP in early pancreatic development (Cebola et al., 2015). The second possible mechanism of Hippo influence on cell proliferation considers the crosstalk between the Hippo signaling pathway and other proliferative signaling, including Notch, WNT/β-catenin, and PI3K/Akt/mTOR signaling. These relationships will be further discussed in the next section.

In addition to TEAD1 regulating the proliferation of pancreatic progenitors, Lee et al. studied TEAD1 function in β cells using two β cell specific Cre drivers, Rip^*Cre*^ which results an early constitutive gene deletion from E15.5, and Mip^*CreER*^ which deletes genes at the time of tamoxifen administration (Lee et al., 2020). The mice with TEAD1 deleted at E15.5 developed early diabetes at 5 weeks of age. Further experiments revealed that deletion of TEAD1 increases the number of β cells in the active phases of the cell cycle in a cell-autonomous way. Mechanistically, Lee et al. found that TEAD1 activates p16^*INK*4a^ in adult β cells to maintain proliferative quiescence. TEAD1 also activates the transcription of critical genes required for maintaining mature β cell function. These data indicate that TEAD1 controls, directly or indirectly, the gene regulatory network critical to maintain β cell functional competence and proliferative quiescence (Lee et al., 2020). Whether the function of TEAD1 in β cells is independent of Hippo pathway remains unknown. Several studies have shown that β cells do not express YAP; however, the expression of TAZ has been found in β cells (Lee et al., 2020). Thus, it will be interesting to find out if deletion of TAZ phenocopies deletion of TEAD1 in β cells.

## The Crosstalk Between Hippo Signaling and the Notch, WNT/β-Catenin, and PI3K/Akt/mTOR Pathway in the Pancreas

The Hippo signaling pathway influences pancreatic cell identity and morphogenesis development by regulating several other signaling pathways. These pathways have been shown to play essential functions during pancreatic organogenesis.

**FIGURE 3:**
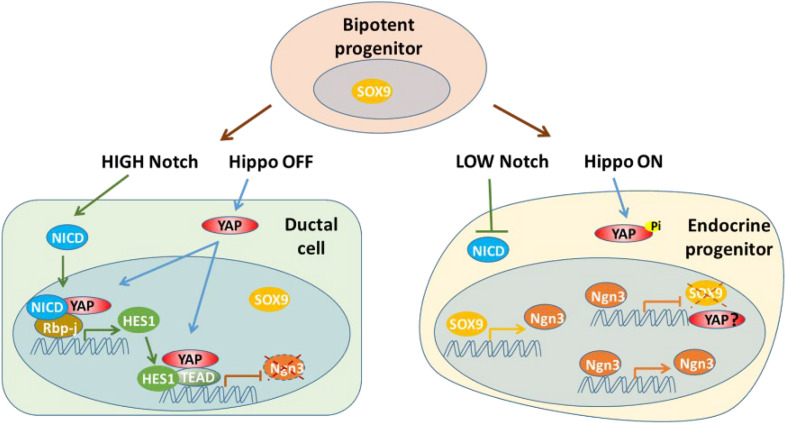
YAP acts as both a transcription activator and repressor with Notch signaling pathway during endocrine development. YAP may be suppressed at transcription level in endocrine progenitor cells.

The Notch signaling pathway has effects on determination of cell fate and normal pancreatic architecture development. During the early stage, Notch signaling helps to maintain proliferation and prevent premature differentiation of pancreatic progenitors into ductal and endocrine cells. Notch signaling regulates pancreatic development through an expression level-dependent manner, instead of a simple “on or off” modality (Murtaugh et al., 2003; Fujikura et al., 2006; Afelik and Jensen, 2013; Li et al., 2015). The Notch pathway affects endocrine linage development by regulating expression of SOX9 and NGN3 via a complex network (Seymour et al., 2007, 2008; Shih et al., 2012). Hes1, one of the target genes and effectors of Notch signaling during pancreatic development, has been found to be a key player in the differentiation of endocrine cells by suppressing Ngn3 expression (Lee et al., 2001). Notch signaling promotes expression of Sox9 to activate expression of Ngn3, which initiates the development of endocrine cells. However, high levels of Notch signaling also induces Hes1 expression, repressing Ngn3 and consequently blocking endocrine cell fate determination. Thus, Notch signaling governs cell fate determination and pancreatic patterning through the actions of Sox9 and Hes1 (Seymour et al., 2007, 2008; Shih et al., 2012). Other studies have found that overexpression of YAP can upregulate Hes1 and Notch1 in YAP1tg pancreata at E15.5, resulting in a phenotype with an expanded ductal compartment. However, this phenotype can be partially rescued by blocking Notch signaling. ChIP-seq analysis has shown that YAP1 acts as an activator of Hes1 transcription to indirectly suppress NGN3, while YAP1-TEAD4-HES1 directly represses the transcription of Ngn3 by specifically binding to the *NGN3* promoter (Gao et al., 2013; Mamidi et al., 2018). Thus, YAP acts as both a transcription activator and repressor during endocrine development (Figure 3).

The WNT/β-catenin signaling pathway also controls pancreatic specification and patterning during different stages, specifically through its effects on proliferation of pancreatic progenitors (Hendley et al., 2015; Scheibner et al., 2019; Sharon et al., 2019). Deletion of WNT related genes in early stages has been found to reduce the MPC pool, which subsequently decreases the amount of both exocrine and endocrine cells (Afelik et al., 2015). Further, the WNT/β-catenin signaling pathway regulates pancreatic epithelial morphogenesis through influencing cell-cell adhesion and regulating the Notch signaling pathway. George et al. suggest that the mechanism causing increased proliferation in the *Mst1/2* null pancreas may be activation of both Wnt/β-catenin and mTOR signaling. They found that *Mst1/2* null pancreata had increased expression of β-catenin, along with β-catenin dephosphorylated to an “active” form, which increased cell proliferation. Expression levels of Wnt signaling target genes, including c-Myc and Tcf1, were also increased in *Mst1/2* null pancreata (Gao et al., 2013). Based on analysis of the regulatory landscape of *in vivo* and *in vitro* MPCs, the enrichment of non-canonical WNT regulators such as FZD2, SFRP5, CELSR2, and VANGL2, suggests an evolutionarily conserved signaling mechanism operating within early pancreatic development (Cebola et al., 2015). It has been shown that LATS1/2 have vital functions to sustain WNT signaling via a YAP-dependent but TEAD-independent pattern in intestinal stem cells (Li et al., 2020). Although this mechanism has not been investigated within the developing pancreas, we expect that the same signaling regulation occurs due to the similarities between pancreatic and intestinal organogenesis.

The mammalian target of Rapamycin (mTOR) integrates signals from both nutrients and growth factors (Elghazi et al., 2017). Inhibition of PKA or mTOR promotes Ngn3-driven β cell regeneration in human T1D islets (Cheng et al., 2017). Within a *Mst1/2* null pancreas, there is an upregulation in expression of phosphorylated S6 and 4E-BP1, indicating that deletion of *Mst1/2* leads to an increase in proliferation via activation of mTOR signaling (George et al., 2012). Akt, an upstream positive regulator of mTOR signaling, has been found to be robustly activated within YapS127A-transduced islets, which have YAP constitutively activated. When treated with mTOR inhibitor rapamycin, the proliferation of β cells which is induced by overexpression of YAP can been blocked in human islets, suggesting that mTOR signaling may regulate mature β cell proliferation downstream of YAP (George et al., 2015). Nonetheless, whether the mTOR signaling pathway is involved in Hippo function during early pancreatic development remains elusive.

## Conclusion and Future Perspective

The Hippo signaling pathway is an essential regulator of pancreatic development, directing cell differentiation and organ morphogenesis. Upstream components of YAP include cell-cell contact and the interaction of the extracellular matrix with integrin α5. However, whether these biological processes influence the function of the Hippo pathway during pancreatic development remains unclear. Components and pathways downstream of YAP include the YAP-TEAD complex and crosstalk with other signaling pathways including Notch, Wnt/β-catenin, and mTOR. Although great progress has been made toward uncovering the mechanisms through which Hippo governs pancreatic development, there are still several components of the pathway that warrant further study.

The upstream kinases of the Hippo pathway, MST1/2 and LATS1/2, have been studied in early pancreatic development. Deletion of either set of kinases stabilizes YAP and TAZ, enabling them to translocate to the nucleus and regulate proliferation, differentiation, and apoptosis. However, *Mst1/2* and *Lats1/2* have only been deleted with Pdx1^*Cre*^ during early pancreatic development, so how these kinases function in the development of specific cell lineages remains largely unknown. Thus, using other Cre lines, such as Ngn3^*Cre*^ or Mip^*Cre*^ (mouse insulin promoter), to study these components in a lineage-specific manner will provide a more comprehensive understanding of the extent to which the Hippo signaling pathway contributes to pancreatic organogenesis.

Pancreatic mesenchymal cells play pivotal roles in pancreas development, mainly via the FGF (fibroblast growth factor) signaling pathway (Lv et al., 2019). Research using single-cell RNA sequencing, *in situ* hybridization, immunofluorescence staining, and genetic lineage tracing, has found that the mesenchymal cells can be identified as 10 transcriptionally distinct populations. Pathway analysis of genes expressed by cells within each population has indicated that Hippo signaling may have important functions within at least some of these cells, suggesting a need for further focus on studying the link between Hippo signaling and the regulation of mesenchymal cells during pancreatic development (Byrnes et al., 2018).

Pancreatic cancer is the third most common cause of cancer-related deaths in the United States. Disruption of Hippo signaling has been found to be associated with pancreatic cancer via promoting pancreatic tumor development and progression, even without mutant Kirsten RAS (KRAS) (Kapoor et al., 2014). Hippo disruption and YAP/TAZ upregulation promotes tumorigenesis through EMT, activation of pancreatic stellate cells, recruitment of immunosuppressive cells, and subsequent resistance to gemcitabine, which is the standard chemotherapeutic agent in pancreatic cancer. The involvement of YAP/TAZ in pancreatic cancer development has been intensively reviewed by others as well (Rooman and Real, 2012; Zhang et al., 2014; Ying et al., 2016; Jiang et al., 2018; Ansari et al., 2019; Eibl and Rozengurt, 2019).

Diabetes mellitus becomes largely intractable once there is loss of β cell function and mass. The Hippo signaling pathway is a key regulator of final organ size as it regulates the balance between cell proliferation and apoptosis. Thus, it has been considered as a potential therapeutic target for increasing β cell proliferation without a change in function (Ardestani and Maedler, 2018). Overexpression of active YAP has been shown to induce β cell proliferation within isolated human islets, but it has no effect on β cell function and functional identity genes. On the other hand, YAP is downregulated in Ngn3 positive endocrine progenitor cells and remains low. We scrutinized the transcriptome of human β cells for expression levels of Hippo pathway components. The data showed that YAP mRNA is low, 0.5 FPKM (Fragments Per Kilobase of transcript per Million mapped reads), TAZ is tenfold higher than YAP at 5.9 FPKM, LATS1 at 10.4, LATS2 at 0.3, MST1 at 2.4, and MST2 at 3.35 (Nica et al., 2013). A study on the deletion of MST1 in mice has shown that MST1 is a critical regulator of β cell apoptosis and functions through direct phosphorylation of PDX1, and that this is a Hippo-independent function (Ardestani et al., 2014). Whether other components of the Hippo pathway play roles in β cell development and function requires further investigation.

The Hippo signaling pathway plays critical roles in maintaining organ size, making it an important pathway to consider manipulating for regenerative medicine (Moya and Halder, 2019). Rosado-Olivieri et al. demonstrated that inhibition of YAP can enhance endocrine progenitor differentiation and result in the generation of improved insulin-secreting cells derived from stem cells (Rosado-Olivieri et al., 2019). On the other hand, even though YAP is not expressed in endocrine cells, two labs have tried to use its pro-proliferation ability to expand insulin producing beta cells (George et al., 2015; Yuan et al., 2016). Here, overexpression of an active form of YAP has been found to greatly induce β cell proliferation in adult human islets. Both labs showed that islets with high levels of YAP expression retain normal gene expression and insulin secretion. However, these experiments must be repeated with islets *in vivo* before attempting to rescue diabetes in human patients. Depending on when YAP is manipulated, inhibiting or activating YAP can both lead to the generation of more insulin-producing cells. It will be interesting to test whether inhibiting YAP during the progenitor stage followed by activating YAP in mature cells will increase the number of insulin-producing cells derived from stem cells (Panciera et al., 2016).

In conclusion, the Hippo signaling pathway has profound impacts on pancreatic development at multiple levels, and its continued roles in tissue homeostasis and tumorigenesis warrant further research into this complex network. Thus, a better understanding of the Hippo pathway’s various stage-dependent contributions within both the developing and mature pancreas will provide insightful knowledge, which can one day be incorporated into the generation of new regenerative and oncologic therapies.

## Author Contributions

YW and PW were responsible for contemplating the concept. YW, PA, MN, LR, JL, and PW were responsible for drafting and editing the manuscript. PW was the guarantor of this work. All authors critically revised and approved the final version of the manuscript.

## Conflict of Interest

The authors declare that the research was conducted in the absence of any commercial or financial relationships that could be construed as a potential conflict of interest.

## References

[B1] Afelik S. Jensen J. (2013). Notch signaling in the pancreas: patterning and cell fate specification. *Wiley Interdiscip. Rev. Dev. Biol.* 2 531–544. 10.1002/wdev.99 24014421

[B2] Afelik S. Pool B. Schmerr M. Penton C. Jensen J. (2015). Wnt7b is required for epithelial progenitor growth and operates during epithelial-to-mesenchymal signaling in pancreatic development. *Dev. Biol.* 399 204–217. 10.1016/j.ydbio.2014.12.031 25576928

[B3] Afelik S. Qu X. Hasrouni E. Bukys M. A. Deering T. Nieuwoudt S. (2012). Notch-mediated patterning and cell fate allocation of pancreatic progenitor cells. *Development* 139 1744–1753. 10.1242/dev.075804 22461559 PMC3328176

[B4] Ansari D. Ohlsson H. Althini C. Bauden M. Zhou Q. Hu D. (2019). The hippo signaling pathway in Pancreatic cancer. *Anticancer Res.* 39 3317–3321. 31262852 10.21873/anticanres.13474

[B5] Arda H. E. Benitez C. M. Kim S. K. (2013). Gene regulatory networks governing pancreas development. *Dev. Cell* 25 5–13. 10.1016/j.devcel.2013.03.016 23597482 PMC3645877

[B6] Ardestani A. Maedler K. (2018). The hippo signaling pathway in Pancreatic beta-cells: functions and regulations. *Endocr. Rev.* 39 21–35. 10.1210/er.2017-00167 29053790

[B7] Ardestani A. Paroni F. Azizi Z. Kaur S. Khobragade V. Yuan T. (2014). MST1 is a key regulator of beta cell apoptosis and dysfunction in diabetes. *Nat. Med.* 20 385–397. 10.1038/nm.3482 24633305 PMC3981675

[B8] Azizoglu D. B. Braitsch C. Marciano D. K. Cleaver O. (2017). Afadin and RhoA control pancreatic endocrine mass via lumen morphogenesis. *Genes Dev.* 31 2376–2390. 10.1101/gad.307637.117 29330353 PMC5795784

[B9] Bastidas-Ponce A. Scheibner K. Lickert H. Bakhti M. (2017). Cellular and molecular mechanisms coordinating pancreas development. *Development* 144 2873–2888. 10.1242/dev.140756 28811309

[B10] Benitez C. M. Goodyer W. R. Kim S. K. (2012). Deconstructing pancreas developmental biology. *Cold Spring Harb. Perspect. Biol.* 4:a012401. 10.1101/cshperspect.a012401 22587935 PMC3367550

[B11] Braitsch C. M. Azizoglu D. B. Htike Y. Barlow H. R. Schnell U. Chaney C. P. (2019). LATS1/2 suppress NFkappaB and aberrant EMT initiation to permit pancreatic progenitor differentiation. *PLoS Biol.* 17:e3000382. 10.1371/journal.pbio.3000382 31323030 PMC6668837

[B12] Byrnes L. E. Wong D. M. Subramaniam M. Meyer N. P. Gilchrist C. L. Knox S. M. (2018). Lineage dynamics of murine pancreatic development at single-cell resolution. *Nat. Commun.* 9:3922. 10.1038/s41467-018-06176-3 PMC6156586 30254276

[B13] Cebola I. Rodriguez-Segui S. A. Cho C. H. Bessa J. Rovira M. Luengo M. (2015). TEAD and YAP regulate the enhancer network of human embryonic pancreatic progenitors. *Nat. Cell Biol.* 17 615–626. 10.1038/ncb3160 25915126 PMC4434585

[B14] Cheng C. W. Villani V. Buono R. Wei M. Kumar S. Yilmaz O. H. (2017). Fasting-mimicking diet promotes Ngn3-driven beta-cell regeneration to reverse diabetes. *Cell* 168 775–788.e712. 28235195 10.1016/j.cell.2017.01.040 PMC5357144

[B15] Cole L. Anderson M. Antin P. B. Limesand S. W. (2009). One process for pancreatic beta-cell coalescence into islets involves an epithelial-mesenchymal transition. *J. Endocrinol.* 203 19–31. 10.1677/joe-09-0072 19608613 PMC3071757

[B16] Dai Y. Jablons D. You L. (2017). Hippo pathway in lung development. *J. Thorac. Dis.* 9 2246–2250. 10.21037/jtd.2017.07.18 28932516 PMC5594189

[B17] Deramaudt T. B. Sachdeva M. M. Wescott M. P. Chen Y. Stoffers D. A. Rustgi A. K. (2006). The PDX1 homeodomain transcription factor negatively regulates the pancreatic ductal cell-specific keratin 19 promoter. *J. Biol. Chem.* 281 38385–38395. 10.1074/jbc.m605891200 17056599

[B18] Eibl G. Rozengurt E. (2019). KRAS, YAP, and obesity in pancreatic cancer: a signaling network with multiple loops. *Semin. Cancer Biol.* 54 50–62. 10.1016/j.semcancer.2017.10.007 29079305 PMC5916582

[B19] Elghazi L. Blandino-Rosano M. Alejandro E. Cras-Meneur C. Bernal-Mizrachi E. (2017). Role of nutrients and mTOR signaling in the regulation of pancreatic progenitors development. *Mol. Metab.* 6 560–573. 10.1016/j.molmet.2017.03.010 28580286 PMC5444096

[B20] Fujikura J. Hosoda K. Iwakura H. Tomita T. Noguchi M. Masuzaki H. (2006). Notch/Rbp-j signaling prevents premature endocrine and ductal cell differentiation in the pancreas. *Cell Metab.* 3 59–65. 10.1016/j.cmet.2005.12.005 16399505

[B21] Fujitani Y. (2017). Transcriptional regulation of pancreas development and beta-cell function [Review]. *Endocr. J.* 64 477–486. 10.1507/endocrj.ej17-0098 28420858

[B22] Gao T. Zhou D. Yang C. Singh T. Penzo-Mendez A. Maddipati R. (2013). Hippo signaling regulates differentiation and maintenance in the exocrine pancreas. *Gastroenterology* 144 1543–1553, 1553.e1. 23454691 10.1053/j.gastro.2013.02.037 PMC3665616

[B23] George N. M. Boerner B. P. Mir S. U. Guinn Z. Sarvetnick N. E. (2015). Exploiting expression of hippo effector, yap, for expansion of functional islet mass. *Mol. Endocrinol.* 29 1594–1607. 10.1210/me.2014-1375 26378466 PMC4627601

[B24] George N. M. Day C. E. Boerner B. P. Johnson R. L. Sarvetnick N. E. (2012). Hippo signaling regulates pancreas development through inactivation of Yap. *Mol. Cell. Biol.* 32 5116–5128. 10.1128/mcb.01034-12 23071096 PMC3510525

[B25] Gomez M. Gomez V. Hergovich A. (2014). The hippo pathway in disease and therapy: cancer and beyond. *Clin. Transl. Med.* 3:22. 10.1186/2001-1326-3-22 PMC4107774 25097725

[B26] Gouzi M. Kim Y. H. Katsumoto K. Johansson K. Grapin-Botton A. (2011). Neurogenin3 initiates stepwise delamination of differentiating endocrine cells during pancreas development. *Dev. Dyn.* 240 589–604. 10.1002/dvdy.22544 21287656

[B27] Gradwohl G. Dierich A. LeMeur M. Guillemot F. (2000). neurogenin3 is required for the development of the four endocrine cell lineages of the pancreas. *Proc. Natl. Acad. Sci. U.S.A.* 97 1607–1611607. 10.1073/pnas.97.4.1607 10677506 PMC26482

[B28] Gu G. Dubauskaite J. Melton D. A. (2002). Direct evidence for the pancreatic lineage: NGN3+ cells are islet progenitors and are distinct from duct progenitors. *Development* 129 2447–2457. 11973276 10.1242/dev.129.10.2447

[B29] Halder G. Johnson R. L. (2011). Hippo signaling: growth control and beyond. *Development* 138 9–22. 10.1242/dev.045500 21138973 PMC2998162

[B30] Hale M. A. Kagami H. Shi L. Holland A. M. Elsasser H. P. Hammer R. E. (2005). The homeodomain protein PDX1 is required at mid-pancreatic development for the formation of the exocrine pancreas. *Dev. Biol.* 286 225–237. 10.1016/j.ydbio.2005.07.026 16126192

[B31] Hendley A. M. Provost E. Bailey J. M. Wang Y. J. Cleveland M. H. Blake D. (2015). p120 Catenin is required for normal tubulogenesis but not epithelial integrity in developing mouse pancreas. *Dev. Biol.* 399 41–53. 10.1016/j.ydbio.2014.12.010 25523391 PMC4868343

[B32] Holden J. K. Cunningham C. N. (2018). Targeting the hippo pathway and cancer through the TEAD family of transcription factors. *Cancers* 10:81. 10.3390/cancers10030081 29558384 PMC5876656

[B33] Hong A. W. Meng Z. Guan K. L. (2016). The Hippo pathway in intestinal regeneration and disease. *Nat. Rev. Gastroenterol. Hepatol.* 13 324–337. 10.1038/nrgastro.2016.59 27147489 PMC5642988

[B34] Jennings R. E. Berry A. A. Strutt J. P. Gerrard D. T. Hanley N. A. (2015). Human pancreas development. *Development* 142 3126–3137. 26395141 10.1242/dev.120063

[B35] Jiang Z. Zhou C. Cheng L. Yan B. Chen K. Chen X. (2018). Inhibiting YAP expression suppresses pancreatic cancer progression by disrupting tumor-stromal interactions. *J. Exp. Clin. Cancer Res.* 37:69. 10.1186/s13046-018-0740-4 PMC5870346 29587800

[B36] Jin K. Xiang M. (2019). Transcription factor Ptf1a in development, diseases and reprogramming. *Cell Mol. Life. Sci.* 76 921–940. 10.1007/s00018-018-2972-z 30470852 PMC11105224

[B37] Kapoor A. Yao W. Ying H. Hua S. Liewen A. Wang Q. (2014). Yap1 activation enables bypass of oncogenic Kras addiction in pancreatic cancer. *Cell* 158 185–197. 10.1016/j.cell.2014.06.003 24954535 PMC4109295

[B38] Kesavan G. Lieven O. Mamidi A. Ohlin Z. L. Johansson J. K. Li W. C. (2014). Cdc42/N-WASP signaling links actin dynamics to pancreatic beta cell delamination and differentiation. *Development* 141 685–696. 10.1242/dev.100297 24449844 PMC3899820

[B39] Kesavan G. Sand F. W. Greiner T. U. Johansson J. K. Kobberup S. Wu X. (2009). Cdc42-mediated tubulogenesis controls cell specification. *Cell* 139 791–801. 10.1016/j.cell.2009.08.049 19914171

[B40] Kim W. Jho E. H. (2018). The history and regulatory mechanism of the hippo pathway. *BMB Rep.* 51 106–118. 10.5483/bmbrep.2018.51.3.022 29397869 PMC5882217

[B41] Larsen H. L. Grapin-Botton A. (2017). The molecular and morphogenetic basis of pancreas organogenesis. *Semin. Cell Dev. Biol.* 66 51–68. 10.1016/j.semcdb.2017.01.005 28089869

[B42] Lee J. C. Smith S. B. Watada H. Lin J. Scheel D. Wang J. (2001). Regulation of the pancreatic pro-endocrine gene neurogenin3. *Diabetes Metab. Res. Rev.* 50 928–936. 10.2337/diabetes.50.5.928 11334435

[B43] Lee J. Liu R. Kim B. S. Zhang Y. Li F. Jagannathan R. (2020). Tead1 reciprocally regulates adult β-cell proliferation and function. *bioRxiv [Preprint]* 10.1101/2020.03.05.979450

[B44] Lee K. Gjorevski N. Boghaert E. Radisky D. C. Nelson C. M. (2011). Snail1, Snail2, and E47 promote mammary epithelial branching morphogenesis. *EMBO J.* 30 2662–2674. 10.1038/emboj.2011.159 21610693 PMC3155296

[B45] Li Q. Sun Y. Jarugumilli G. K. Liu S. Dang K. Cotton J. L. (2020). Lats1/2 sustain intestinal stem cells and wnt activation through TEAD-dependent and independent transcription. *Cell Stem Cell* 26 675–692.e678. 32259481 10.1016/j.stem.2020.03.002 PMC7310193

[B46] Li X. Y. Zhai W. J. Teng C. B. (2015). Notch signaling in pancreatic development. *Int. J. Mol. Sci.* 17:48. 10.3390/ijms17010048 26729103 PMC4730293

[B47] Liu J. Gao M. Nipper M. Deng J. Sharkey F. E. Johnson R. L. (2019). Activation of the intrinsic fibroinflammatory program in adult pancreatic acinar cells triggered by hippo signaling disruption. *PLoS Biol.* 17:e3000418. 10.1371/journal.pbio.3000418 31513574 PMC6742234

[B48] Lof-Ohlin Z. M. Nyeng P. Bechard M. E. Hess K. Bankaitis E. Greiner T. U. (2017). EGFR signalling controls cellular fate and pancreatic organogenesis by regulating apicobasal polarity. *Nat. Cell Biol.* 19 1313–1325. 10.1038/ncb3628 29058721

[B49] Lv Y. Q. Wu J. Li X. K. Zhang J. S. Bellusci S. (2019). Role of FGF10/FGFR2b signaling in mouse digestive tract development, repair and regeneration following injury. *Front. Cell Dev. Biol.* 7:326. 10.3389/fcell.2019.00326 PMC6914673 31921841

[B50] Magenheim J. Klein A. M. Stanger B. Z. Ashery-Padan R. Sosa-Pineda B. Gu G. (2011). Ngn3(+) endocrine progenitor cells control the fate and morphogenesis of pancreatic ductal epithelium. *Dev. Biol.* 359 26–36. 10.1016/j.ydbio.2011.08.006 21888903 PMC3746519

[B51] Magnuson M. A. Osipovich A. B. (2013). Pancreas-specific Cre driver lines and considerations for their prudent use. *Cell Metab.* 18 9–20. 10.1016/j.cmet.2013.06.011 23823474 PMC3732107

[B52] Mamidi A. Prawiro C. Seymour P. A. de Lichtenberg K. H. Jackson A. Serup P. (2018). Mechanosignalling via integrins directs fate decisions of pancreatic progenitors. *Nature* 564 114–118. 10.1038/s41586-018-0762-2 30487608

[B53] Marty-Santos L. Cleaver O. (2016). Pdx1 regulates pancreas tubulogenesis and E-cadherin expression. *Development* 143 101–112. 10.1242/dev.126755 26657766 PMC4725206

[B54] Maugeri-Sacca M. De Maria R. (2018). The Hippo pathway in normal development and cancer. *Pharmacol. Ther.* 186 60–72. 10.1016/j.pharmthera.2017.12.011 29305295

[B55] McDonald E. Li J. Krishnamurthy M. Fellows G. F. Goodyer C. G. Wang R. (2012). SOX9 regulates endocrine cell differentiation during human fetal pancreas development. *Int. J. Biochem. Cell Biol.* 44 72–83. 10.1016/j.biocel.2011.09.008 21983268

[B56] McGrath P. S. Watson C. L. Ingram C. Helmrath M. A. Wells J. M. (2015). The basic helix-loop-helix transcription factor NEUROG3 is required for development of the human endocrine pancreas. *Diabetes Metab. Res. Rev.* 64 2497–2505. 10.2337/db14-1412 25650326 PMC4477351

[B57] Mo J. S. Park H. W. Guan K. L. (2014). The Hippo signaling pathway in stem cell biology and cancer. *EMBO Rep.* 15 642–656. 10.15252/embr.201438638 24825474 PMC4197875

[B58] Morris H. T. Machesky L. M. (2015). Actin cytoskeletal control during epithelial to mesenchymal transition: focus on the pancreas and intestinal tract. *Br. J. Cancer* 112 613–620. 10.1038/bjc.2014.658 25611303 PMC4333498

[B59] Moya I. M. Halder G. (2019). Hippo-YAP/TAZ signalling in organ regeneration and regenerative medicine. *Nat. Rev. Mol. Cell Biol.* 20 211–226. 10.1038/s41580-018-0086-y 30546055

[B60] Murtaugh L. C. Stanger B. Z. Kwan K. M. Melton D. A. (2003). Notch signaling controls multiple steps of pancreatic differentiation. *Proc. Natl. Acad. Sci. U.S.A.* 100 14920–14925. 10.1073/pnas.2436557100 14657333 PMC299853

[B61] Nantie L. B. Young R. E. Paltzer W. G. Zhang Y. Johnson R. L. Verheyden J. M. (2018). Lats1/2 inactivation reveals Hippo function in alveolar type I cell differentiation during lung transition to air breathing. *Development* 145:dev.163105. 10.1242/dev.163105 PMC6240317 30305289

[B62] Nica A. C. Ongen H. Irminger J. C. Bosco D. Berney T. Antonarakis S. E. (2013). Cell-type, allelic, and genetic signatures in the human pancreatic beta cell transcriptome. *Genome Res.* 23 1554–1562. 10.1101/gr.150706.112 23716500 PMC3759730

[B63] Noce V. Battistelli C. Cozzolino A. M. Consalvi V. Cicchini C. Strippoli R. (2019). YAP integrates the regulatory Snail/HNF4alpha circuitry controlling epithelial/hepatocyte differentiation. *Cell Death Dis.* 10:768. 10.1038/s41419-019-2000-8 PMC6787001 31601778

[B64] Panciera T. Azzolin L. Fujimura A. Di Biagio D. Frasson C. Bresolin S. (2016). Induction of expandable tissue-specific stem/progenitor cells through transient expression of YAP/TAZ. *Cell Stem Cell* 19 725–737. 10.1016/j.stem.2016.08.009 27641305 PMC5145813

[B65] Piccolo S. Dupont S. Cordenonsi M. (2014). The biology of YAP/TAZ: hippo signaling and beyond. *Physiol. Rev.* 94 1287–1312. 10.1152/physrev.00005.2014 25287865

[B66] Rooman I. Real F. X. (2012). Pancreatic ductal adenocarcinoma and acinar cells: a matter of differentiation and development? *Gut* 61 449–458. 10.1136/gut.2010.235804 21730103

[B67] Rosado-Olivieri E. A. Anderson K. Kenty J. H. Melton D. A. (2019). YAP inhibition enhances the differentiation of functional stem cell-derived insulin-producing beta cells. *Nat. Commun.* 10:1464. 10.1038/s41467-019-09404-6 PMC6443737 30931946

[B68] Rukstalis J. M. Habener J. F. (2007). Snail2, a mediator of epithelial-mesenchymal transitions, expressed in progenitor cells of the developing endocrine pancreas. *Gene Expr. Patterns* 7 471–479. 10.1016/j.modgep.2006.11.001 17185046 PMC2698037

[B69] Rukstalis J. M. Habener J. F. (2009). Neurogenin3: a master regulator of pancreatic islet differentiation and regeneration. *Islets* 1 177–184. 10.4161/isl.1.3.9877 21099270

[B70] Santucci M. Vignudelli T. Ferrari S. Mor M. Scalvini L. Bolognesi M. L. (2015). The hippo pathway and YAP/TAZ-TEAD protein-protein interaction as targets for regenerative medicine and cancer treatment. *J. Med. Chem.* 58 4857–4873. 10.1021/jm501615v 25719868

[B71] Scheibner K. Bakhti M. Bastidas-Ponce A. Lickert H. (2019). Wnt signaling: implications in endoderm development and pancreas organogenesis. *Curr. Opin. Cell Biol.* 61 48–55. 10.1016/j.ceb.2019.07.002 31377680

[B72] Seymour P. A. (2014). Sox9: a master regulator of the pancreatic program. *Rev. Diabet Stud.* 11 51–83. 10.1900/rds.2014.11.51 25148367 PMC4295800

[B73] Seymour P. A. Freude K. K. Dubois C. L. Shih H. P. Patel N. A. Sander M. (2008). A dosage-dependent requirement for Sox9 in pancreatic endocrine cell formation. *Dev. Biol.* 323 19–30. 10.1016/j.ydbio.2008.07.034 18723011 PMC2879081

[B74] Seymour P. A. Freude K. K. Tran M. N. Mayes E. E. Jensen J. Kist R. (2007). SOX9 is required for maintenance of the pancreatic progenitor cell pool. *Proc. Natl. Acad. Sci. U.S.A.* 104 1865–1870. 10.1073/pnas.0609217104 17267606 PMC1794281

[B75] Sharon N. Vanderhooft J. Straubhaar J. Mueller J. Chawla R. Zhou Q. (2019). Wnt signaling separates the progenitor and endocrine compartments during pancreas development. *Cell Rep.* 27 2281–2291.e5. 31116975 10.1016/j.celrep.2019.04.083 PMC6933053

[B76] Shih H. P. Kopp J. L. Sandhu M. Dubois C. L. Seymour P. A. Grapin-Botton A. (2012). A Notch-dependent molecular circuitry initiates pancreatic endocrine and ductal cell differentiation. *Development* 139 2488–2499. 10.1242/dev.078634 22675211 PMC3383226

[B77] Shih H. P. Wang A. Sander M. (2013). Pancreas organogenesis: from lineage determination to morphogenesis. *Annu. Rev. Cell Dev. Biol.* 29 81–105. 10.1146/annurev-cellbio-101512-122405 23909279

[B78] Skibinski A. Breindel J. L. Prat A. Galván P. Smith E. Rolfs A. (2014). The Hippo transducer TAZ interacts with the SWI/SNF complex to regulate breast epithelial lineage commitment. *Cell Rep.* 6 1059–1072. 10.1016/j.celrep.2014.02.038 24613358 PMC4011189

[B79] van Soldt B. J. Qian J. Li J. Tang N. Lu J. Cardoso W. V. (2019). Yap and its subcellular localization have distinct compartment-specific roles in the developing lung. *Development* 146:dev.175810. 10.1242/dev.175810 PMC6526715 30944105

[B80] Villasenor A. Chong D. C. Cleaver O. (2008). Biphasic Ngn3 expression in the developing pancreas. *Dev. Dyn.* 237 3270–3279. 10.1002/dvdy.21740 18924236 PMC2597057

[B81] Villasenor A. Marty-Santos L. Dravis C. Fletcher P. Henkemeyer M. Cleaver O. (2012). EphB3 marks delaminating endocrine progenitor cells in the developing pancreas. *Dev. Dyn.* 241 1008–1019. 10.1002/dvdy.23781 22434763 PMC3328632

[B82] Vinogradova T. V. Sverdlov E. D. (2017). PDX1: a unique pancreatic master regulator constantly changes its functions during embryonic development and progression of pancreatic cancer. *Biochemistry (Mosc).* 82 887–893. 10.1134/S000629791708003X 28941456

[B83] Wang J. Liu S. Heallen T. Martin J. F. (2018). The Hippo pathway in the heart: pivotal roles in development, disease, and regeneration. *Nat. Rev. Cardiol.* 15 672–684. 10.1038/s41569-018-0063-3 30111784

[B84] Wang S. Yan J. Anderson D. A. Xu Y. Kanal M. C. Cao Z. (2010). Neurog3 gene dosage regulates allocation of endocrine and exocrine cell fates in the developing mouse pancreas. *Dev. Biol.* 339 26–37. 10.1016/j.ydbio.2009.12.009 20025861 PMC2824035

[B85] Wu N. Nguyen Q. Wan Y. Zhou T. Venter J. Frampton G. A. (2017). The Hippo signaling functions through the Notch signaling to regulate intrahepatic bile duct development in mammals. *Lab. Invest.* 97 843–853. 10.1038/labinvest.2017.29 28581486 PMC5901959

[B86] Ying H. Dey P. Yao W. Kimmelman A. C. Draetta G. F. Maitra A. (2016). Genetics and biology of pancreatic ductal adenocarcinoma. *Genes Dev.* 30 355–385. 26883357 10.1101/gad.275776.115 PMC4762423

[B87] Yuan T. Rafizadeh S. Azizi Z. Lupse B. Gorrepati K. D. D. Awal S. (2016). Proproliferative and antiapoptotic action of exogenously introduced YAP in pancreatic β cells. *JCI Insight* 1:e86326. 10.1172/jci.insight.86326 PMC5085606 27812538

[B88] Zhang W. Nandakumar N. Shi Y. Manzano M. Smith A. Graham G. (2014). Downstream of mutant KRAS, the transcription regulator YAP is essential for neoplastic progression to pancreatic ductal adenocarcinoma. *Sci. Signal.* 7:ra42. 10.1126/scisignal.2005049 24803537 PMC4175524

[B89] Zhang Z. W. Men T. Feng R. C. Li Y. C. Zhou D. Teng C. B. (2013). miR-375 inhibits proliferation of mouse pancreatic progenitor cells by targeting YAP1. *Cell Physiol. Biochem.* 32 1808–1817. 10.1159/000356614 24356001

[B90] Zheng Y. Pan D. (2019). The hippo signaling pathway in development and disease. *Dev. Cell* 50 264–282. 10.1016/j.devcel.2019.06.003 31386861 PMC6748048

[B91] Zhou Q. Melton D. A. (2018). Pancreas regeneration. *Nature* 557 351–358. 29769672 10.1038/s41586-018-0088-0 PMC6168194

